# Arteriovenous Malformation Presenting With Bone Destruction of the Proximal Radius: A Case Report

**DOI:** 10.7759/cureus.47336

**Published:** 2023-10-19

**Authors:** Ameer Sayed, Hatem Alharbi, Mohammed K Aldawsari, Haleema S Almonaye, Hatim Mohammed Alshareef, Ahmed N Aljedani

**Affiliations:** 1 Department of Orthopaedic Surgery, King Fahad Armed Forces Hospital, Jeddah, SAU; 2 Department of Orthopaedic Surgery, King Saud Bin Abdulaziz University for Health Sciences College of Medicine, Jeddah, SAU; 3 Department of Orthopaedic Surgery, Batterjee Medical College, Jeddah, SAU

**Keywords:** proximal radius mass, proximal radius pain, osteolytic lesion, bone erosion, arteriovenous malformation

## Abstract

Erosive bony lesions are a frequent manifestation of numerous etiologies, spanning from malignancy and metabolic disorders to chronic inflammatory conditions like sarcoidosis. However, arteriovenous malformations (AVM) are a less prevalent etiology for this condition. The presentation of erosive bony lesions is diverse, influenced by factors such as age, gender, size, and extent of the lesion. Multiple imaging modalities are employed to achieve a diagnosis, including plain radiograph, Doppler ultrasound, computed tomography, angiography, and magnetic resonance imaging.

## Introduction

Bone erosion is a condition characterized by localized bone loss, also known as osteolysis. This occurs when bone resorption by osteoclasts exceeds bone formation by osteoblasts. Radiologically, erosive bone lesions appear as a break in cortical bone, with the destruction of the natural barrier between the extraskeletal and tissue and the bone marrow compartment [1]. Numerous pathological processes can lead to this condition, including malignancy, metabolic processes such as hyperparathyroidism, chronic inflammatory disease like sarcoidosis, and arteriovenous malformation [1,2]. In this case study, we present a 46-year-old female patient who experienced chronic on-and-off vague pain in her left forearm for a decade. After conducting a thorough investigation, we discovered the presence of a large AVM in her left elbow region that extended to the proximal forearm. It is important to note that AVM can also contribute to the development of bone erosion and, therefore, must be considered a potential etiological factor.

## Case presentation

This case concerns a 46-year-old female who, to the best of her knowledge, had not suffered from any prior medical conditions. Despite visiting numerous hospitals seeking medical attention, she presented to our orthopedic clinic with complaints of chronic intermittent vague left forearm pain for over 10 years. Although the pain was initially treated conservatively as muscle strain, there was no incident leading to the onset of pain. There were no indications of fever, night sweats, weight loss, or other systemic manifestations. Preceding her appointment at the orthopedic clinic, the patient presented to the emergency department with sudden worsening of her left shoulder pain associated with numbness. The patient was managed conservatively with analgesia and provided an appointment with our orthopedic clinic for further management. Upon presenting to the orthopedic clinic, the patient underwent a physical examination that revealed she had swelling over the proximal forearm, as well as engorged tortuous disfiguring veins of the left upper limb extending from the left shoulder to the left hand. Additionally, a pulsating cystic swelling was observed over the proximal forearm, with a positive thrill present. The patient also displayed restricted supination of the left forearm, although full pronation was possible. Mild pain was reported when flexing the left elbow and at 10 degrees extension in both elbows. Finally, mild tenderness was presented upon palpation of the proximal left forearm, but the distal neurovascular examination was intact.

The patient’s laboratory results are listed in Table 1.

**Table 1:** The patient’s laboratory results ESR: erythrocyte sedimentation rate; CRP: C-reactive protein

Labs	Patient's value	Normal range
WBC count	4.13 10^9/L	3.3 - 10.8
Hemoglobin level	122 G/L	120 - 160
ESR	42 Mm/Hr	0 - 14
CRP	<1 MG/L	-5

**Figure 1:**
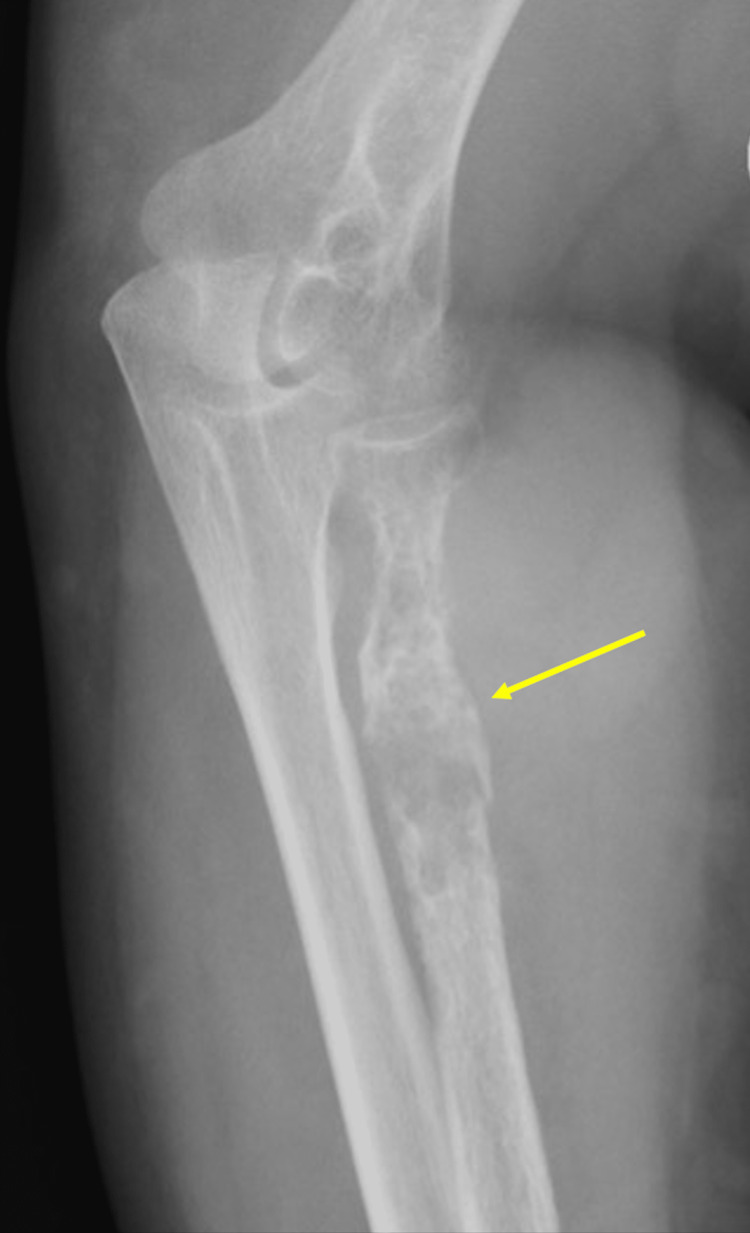
Lateral X-ray of the left elbow and proximal forearm showing aggressive bone erosion

**Figure 2:**
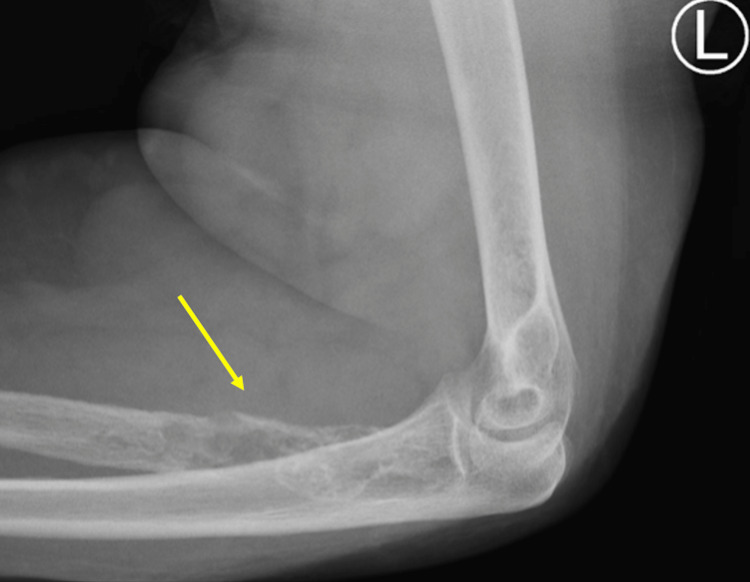
Lateral X-ray of the left elbow and proximal forearm showing aggressive bone erosion

**Figure 3:**
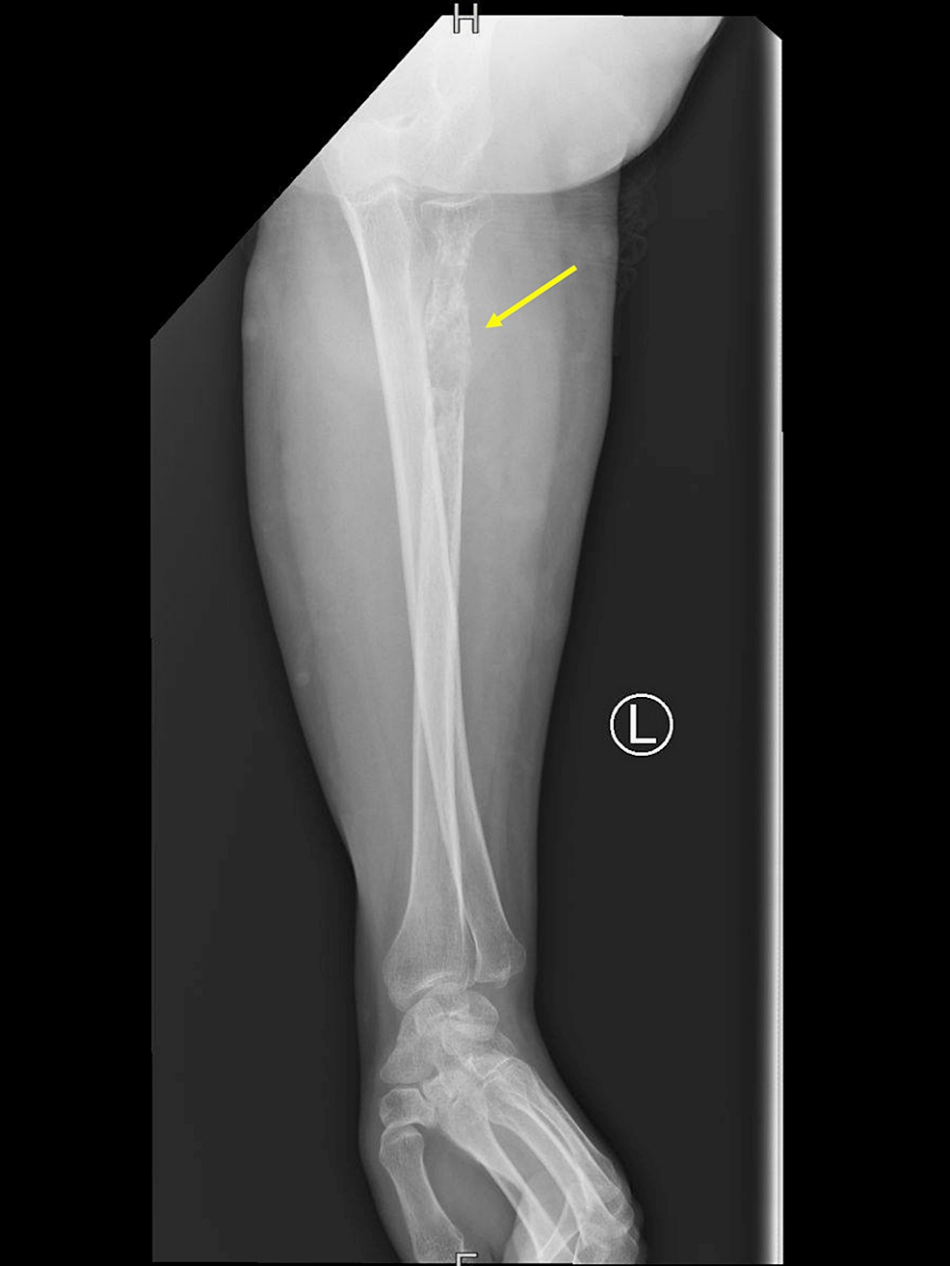
Anteroposterior (AP) X-ray view of the left forearm showing an erosive bony lesion

An aggressive osteopathic lesion of the proximal one-third of the radius was observed through X-ray imaging of the left forearm and elbow (Figures 1-3).

**Figure 4:**
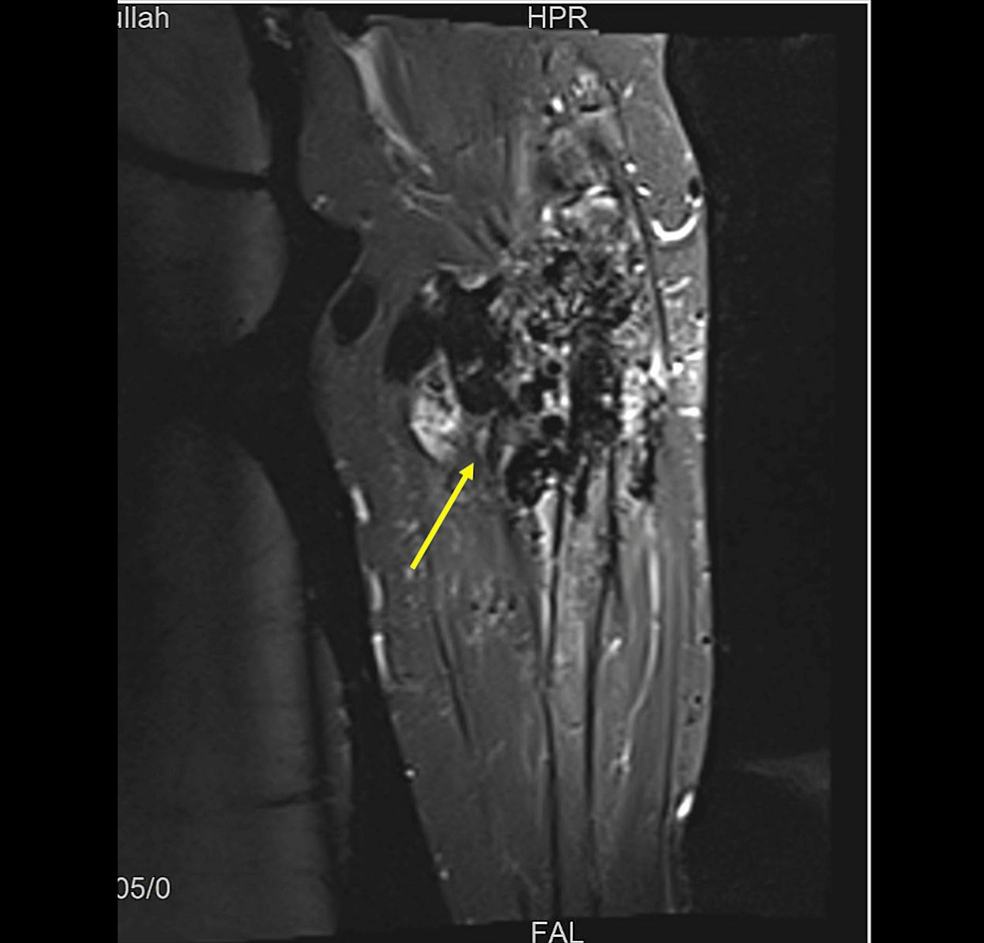
Magnetic resonance imaging (MRI) showing prominent forearm vessels

**Figure 5:**
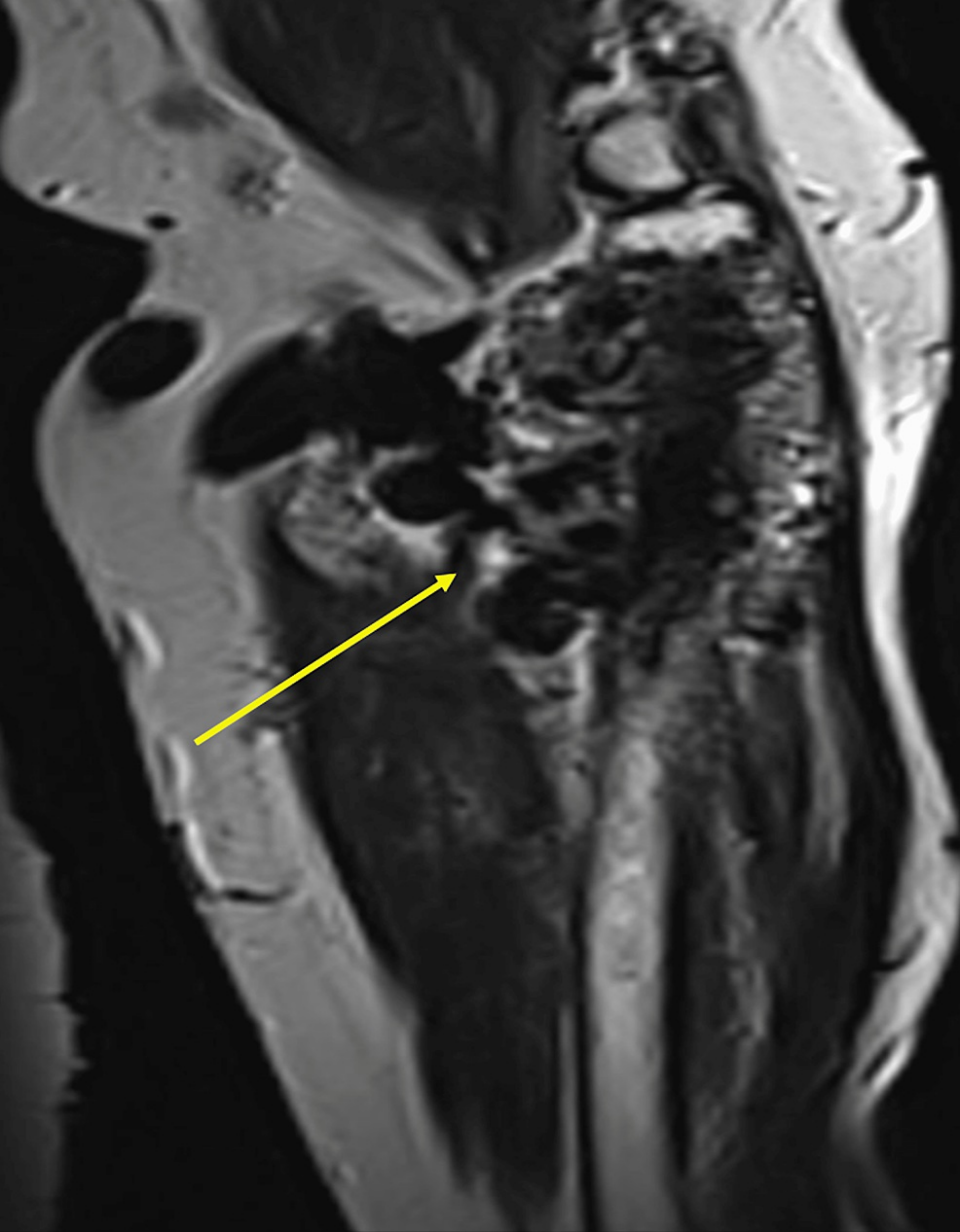
Magnetic resonance imaging (MRI) showing infiltration of the proximal radius bone

Further diagnostic testing through ultrasound (US) showed tortuous and increased diameter of superficial cephalomedullary and basilic veins measured more than 1 cm, with aliasing and turbulent flow on color and spectral Doppler analysis. However, no evidence of thrombosis was detected. No obvious soft tissue masses, collections, or subcutaneous edema were noted. To screen for malignancy, computed tomography imaging of the chest, abdomen, and pelvis was conducted, yielding unremarkable results. Meanwhile, MRI revealed significant dilation and tortuosity with the maximum diameter measuring up to 2.8 cm at the elbow level. Multiple prominent and tortuous arm and forearm vascular structures are seen mostly as flow-void structures at T1 and T2, with extensive forearm infiltration and extension to the proximal radius shaft and metadiaphysis, leading to erosion. However, no involvement of the ulna or distal humerus was noted in the imaging findings. These results are highly indicative of peripheral vascular malformation (Figures 4, 5).

**Figure 6:**
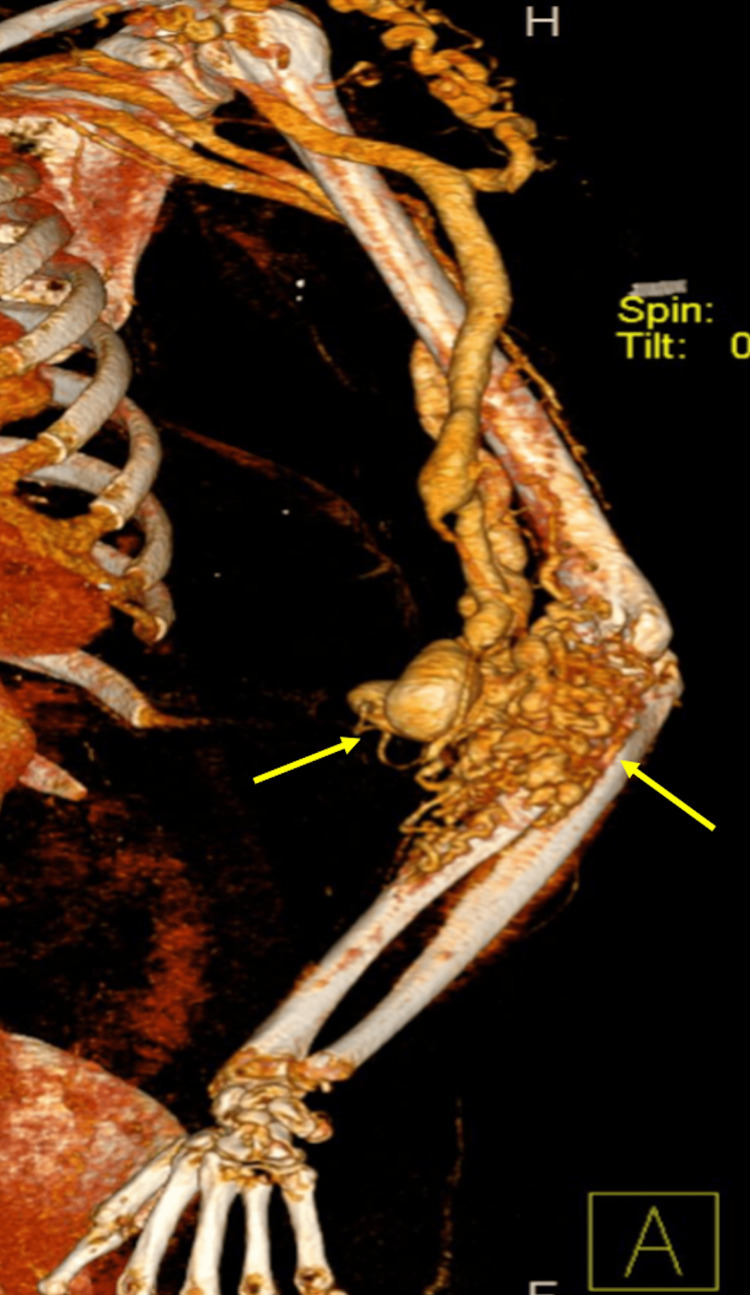
3-dimensional computerized tomography (CT) scan showing revealing tortuous arm and forearm vessels

**Figure 7:**
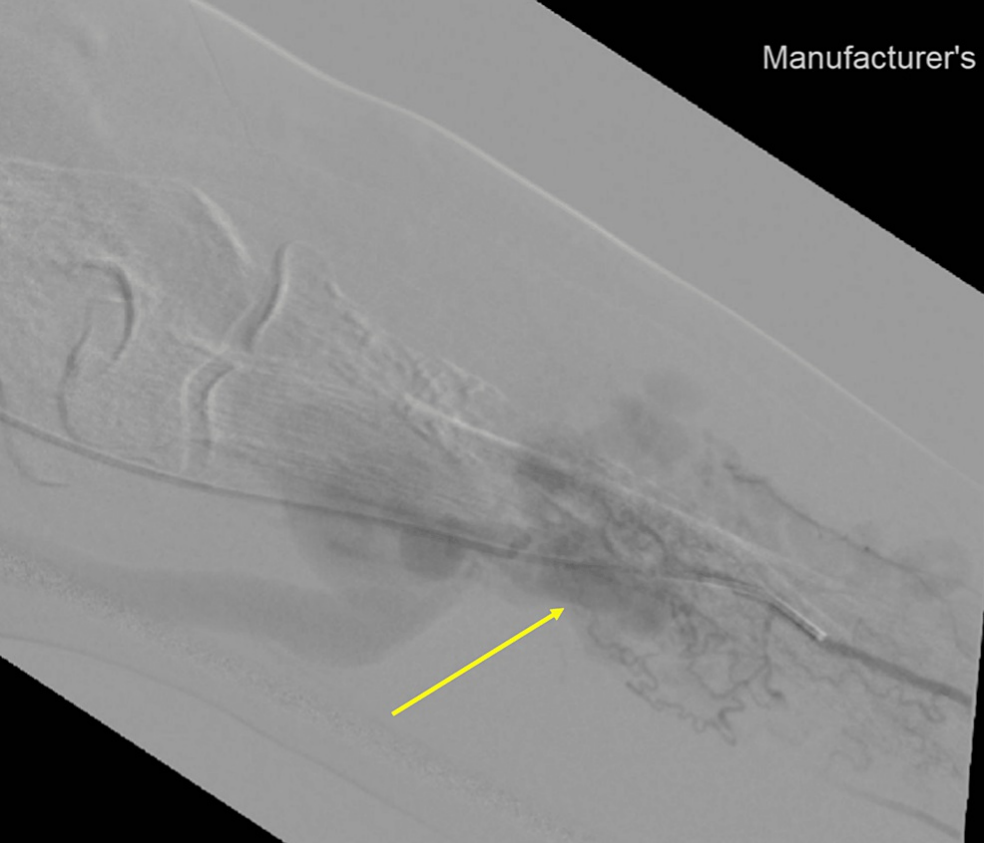
Angiographic image of the vessels of the left forearm

A CT angiography detected a significant arteriovenous malformation nidus in the left elbow to proximal forearm region measuring 4.7X7.3X9.8 cm in maximum dimension with direct arterial feeders from the brachial artery, venous drainage through direct and indirect tributaries into the cephalic, basilic, and brachial veins, tortuous venous collaterals seen at the right shoulder and supraclavicular region draining into the cephalic vein. Cortical erosion and infiltration of the proximal radial shaft (Figures 6, 7).

The vascular surgery team was consulted to determine the optimum management for this patient. They recommended embolization by interventional radiology, but due to the high risk associated with the lesion, catheterization attempts were unsuccessful. Consequently, it was recommended that the patient be referred to a specialized center for the management of complex vascular malformation.

## Discussion

AVMs are commonly accounted for as a rare congenital vascular lesion that typically results from faulty embryogenesis leading to an abnormal connection between the arteries and veins, creating a central nidus without intervening capillary bed. This condition is associated with various complications such as destruction of the adjacent soft tissues and bones, venous hypertension, and heart failure among others [3,4]. AVM might be associated with syndromes such as Kippel-Trénaunay syndrome, Parkes-Weber syndrome, and Stewart-Bluefarb syndrome [5]. Additionally, other less frequent etiologies of AVM include trauma, degenerative vascular disease, and iatrogenic causes [6]. Intracranial AVM represents the most common site of occurrence [4]. Other reported locations for AVM involvement are bones, especially the extremities with a high incidence reaching 30%. However, it also occurs in the pelvis, mid-face, and oral cavity [2]. The most commonly involved area of the skeletal system is the diaphysis of long bones [7]. The clinical presentation of AVM is influenced by several factors, including age, sex, size, and the extent of the malformation [5]. These malformations are usually present since birth, gradually progressing over time, and later on, may become evident presenting with pain, a mass that could be pulsatile, skin ulceration, bleeding, venous varicosities, functional limitation, limb length discrepancy (LLD), osteolytic bone lesions, and pathological fractures [2,5,8]. To ensure an accurate diagnosis, a diligent history and physical examination should be conducted alongside various investigations [5]. Plain radiographs may reveal bone erosion, sclerotic or periosteal reaction, cortical scalloping, or pathological fractures [9]. For detecting and assessing the extent of AVM lesions, US, contrast-enhanced CT imaging, and MRI are highly useful. Although, arteriography remains the imaging modality of choice to evaluate AVM [5,8,9]. The management of AVM necessitates a multidisciplinary approach, and treatment options range from conservative measures, such as fitted pressure garments, analgesia, systemic corticosteroids, embolization, radiation, sclerosing agents, to more invasive surgical resection, or possibly a combination of mentioned modalities [2,7,10].

## Conclusions

Bone erosion is a complex condition that arises from an imbalance between bone resorption and formation. Its pathogenesis can be multifactorial and may involve various disease processes, including AVM, which can often be misdiagnosed as other malicious lesions like tumors. Therefore, meticulous imaging is paramount in reaching a definitive diagnosis and avoiding unwarranted biopsy or surgery. Despite the complexity of the condition, there is still some debate over the optimal treatment method, with less invasive image-guided and interventional treatments being the most commonly employed strategies to date. Nonetheless, the advancement of medical technology continues to aid in finding the most effective approach to treating bone erosion. Overall, it is essential to approach bone erosion in a holistic manner, taking into account the multifactorial nature of its pathogenesis and adopting a personalized treatment approach based on the patient’s specific needs. By doing so, healthcare professionals can provide the best possible care for those affected by this complex condition.
